# Neural representation of perceived race mediates the opposite relationship between subcomponents of self-construals and racial outgroup punishment

**DOI:** 10.1093/cercor/bhad157

**Published:** 2023-05-04

**Authors:** Yuqing Zhou, Wenxin Li, Tianyu Gao, Xinyue Pan, Shihui Han

**Affiliations:** CAS Key Laboratory of Behavioral Science, Institute of Psychology, Chinese Academy of Sciences, 16 Lincui Road, Beijing 100101, China; Department of Psychology, University of Chinese Academy of Sciences, Beijing 100049, China; School of Psychological and Cognitive Sciences, Beijing Key Laboratory of Behavior and Mental Health, PKU-IDG/McGovern Institute for Brain Research, Peking University, 52 Haidian Road, Beijing 100080, China; Department of Psychology, Faculty of Arts and Sciences, Beijing Normal University, Zhuhai 519087, China; School of Psychological and Cognitive Sciences, Beijing Key Laboratory of Behavior and Mental Health, PKU-IDG/McGovern Institute for Brain Research, Peking University, 52 Haidian Road, Beijing 100080, China; School of Psychological and Cognitive Sciences, Beijing Key Laboratory of Behavior and Mental Health, PKU-IDG/McGovern Institute for Brain Research, Peking University, 52 Haidian Road, Beijing 100080, China

**Keywords:** EEG, interdependence, punishment decisions, intergroup conflicts, race

## Abstract

Outgroup aggression characterizes intergroup conflicts in human societies. Previous research on relationships between cultural traits and outgroup aggression behavior showed inconsistent results, leaving open questions regarding whether cultural traits predict individual differences in outgroup aggression and related neural underpinnings. We conducted 2 studies to address this issue by collecting self-construal scores, EEG signals in response to Asian and White faces with painful or neutral expressions, and decisions to apply electric shocks to other-race individuals in a context of interracial conflict. We found that interdependent self-construals were well explained by 2 subcomponents, including esteem for group (EG) and relational interdependence (RI), which are related to focus on group collectives and harmonious relationships, respectively. Moreover, EG was positively associated with the decisions to punish racial outgroup targets, whereas RI was negatively related to the decisions. These opposite relationships were mediated by neural representations of perceived race at 120–160 ms after face onset. Our findings highlight the multifaceted nature of interdependent self-construal and the key role of neural representations of race in mediating the relationships of different subcomponents of cultural traits with racial outgroup punishment decisions in a context of interracial conflict.

## Introduction

People tend to punish others who are responsible for an injury (Elshout et al. 2015; Jackson et al. 2019). This behavioral tendency is particularly salient during intergroup conflicts (Gelfand et al. 2012; Amodio and Cikara 2021) and plays a causal role in homicides and transgenerational conflicts (Kopsaj 2016; Jackson et al. 2019). It is now known that punishment decisions during intergroup conflicts are mediated by specific neurobiological mechanisms (e.g. Han et al. 2020, 2021) and may vary across individuals or social groups due to their social–cultural experiences (Gelfand et al. 2012). However, it remains unclear whether cultural traits are associated with punishment decisions on outgroup in a context of intergroup conflicts and, if such associations exist, what are the underlying brain mechanisms.

It has been assumed that outgroup aggression in intergroup conflict is more likely to occur in collectivistic than individualistic societies (Gelfand et al. 2012). However, behavioral research also showed evidence that people from collectivistic societies were more in favor of and expressed less prejudice toward outgroups (Gouveia 2011). In addition, recent behavioral and brain imaging research revealed that outgroup derogation in empathy and altruistic behaviors exists in different cultural samples, which endorses either collectivism or individualism (Xu et al. 2009; Han 2018; Romano et al. 2021). The seemingly inconsistency between theoretical views and empirical findings requires to be clarified by examining the relationships between a specific cultural trait and outgroup punishments during intergroup conflicts and intermediate brain mechanisms.

Interdependence refers to a cultural trait that depicts how an individual is sensitive to information related to significant others during social interactions. Interdependence as a key characteristic of self-construals is associated with variations in cognition, emotion, behavior, and brain activities across different cultural samples and individuals (Markus and Kitayama 1991; Nisbett and Masuda 2003; Han and Northoff 2008; Cross et al. 2011; Han et al. 2013; Han and Ma 2015). While early research measured interdependence as a single cultural trait (Singelis 1994), recent studies have shown evidence that it consists of multiple subcomponents (Oyserman et al. 2002; Hardin et al. 2004; Hardin 2006) that account for cultural group differences in social emotion (Hardin 2006). For example, a factor analysis of self-construal scores (Singelis 1994) obtained from an American sample identified 2 subcomponents of interdependence, including esteem for group (EG) and relational interdependence (RI) (Hardin et al. 2004). These 2 subcomponents mapped closely to the relative focus on group collectives and harmonious relationships, respectively (Cross et al. 2011). If the interest of group collectives leads to an increased tendency to harm outgroup during intergroup conflicts (Markus and Kitayama 2010; Gelfand et al. 2012), whereas the pursuit of harmonious relationships facilitates egalitarian views of social relationships (Gouveia 2011), EG and RI may have “opposite” relationships with outgroup punishments in the context of intergroup conflicts.

The current work tested this prediction by examining the relationships between interdependence and punishment decision-making pertaining to outgroups in the context of intergroup conflicts. We collected questionnaire, behavioral, and large sample EEG measures that allowed estimation of the relationships between interdependence and outgroup punishment and the underlying brain mechanisms. Two possible neural mechanisms may mediate the association between interdependence and outgroup punishment during intergroup conflicts. First, as an interdependent schema of self organizes behavior in reference to the thoughts and feelings of close others, which leads to significant ingroup–outgroup distinction (Markus and Kitayama 2010), and the enhanced group categorization processes are associated with decreased altruistic behavior toward racial outgroups (Zhou et al. 2020), brain activities underlying group perception may mediate the effects of interdependent self-construals on outgroup punishments during intergroup conflicts. Alternatively, since empathy for one’s suffering is related to punishment decisions (Batson et al. 2002; Molenberghs et al. 2014; Pfattheicher et al. 2019; Han et al. 2020), and interdependence affects the empathic accuracy of understanding and sharing others’ emotional states (Atkins et al. 2016), it is likely that brain activities underlying empathy may link the relationship between interdependent self-construals and outgroup punishment.

Racial identities of faces are spontaneously encoded in the brain (Zhou et al. 2020) and perceived race is a byproduct of cognitive processes that evolved to detect ingroup/outgroup (Kurzban et al. 2001). In addition, perceived race results in racial ingroup biases in empathy and social behavior (Sheng and Han 2012; Han 2018). Therefore, the current study sought to disentangle the neural activities underlying race perception and empathy to clarify whether these brain activities are engaged to mediating associations between interdependent self-construals and outgroup punishment in a context of conflicts between individuals of different races. We collected questionnaires, behavioral, and EEG measures in 2 large Chinese samples. Specifically, we were interested in punishment behavior pertaining to outgroups during intergroup conflicts. We first tested and validated the 2 subcomponents of interdependence, i.e. EG and RI, in 2 Chinese samples in studies 1 and 2 (*n* = 2,297 and 676), similar to the findings of previous research that tested American samples (Hardin et al. 2004). We then assessed the relationships between the subcomponents of interdependence and racial outgroup punishments by asking participants in study 2 to make punishment decisions toward racial outgroups in the context of interracial conflicts due to painful stimulations.

To clarify whether the brain systems involved in race perception or empathy mediate the relationships between EG/RI and racial outgroup punishments, we collected a large sample of EEG signals (*n* = 676) in response to painful and neutral expressions of Asian and White faces. Previous research using this paradigm showed that the neural responses elicited by painful expression associated with the subjective feeling of empathy (Sheng and Han 2012), and reflected the activations in the empathic neural network (Sheng et al. 2014; Zhou and Han 2021), making the paradigm suitable to examine the neural representation of race perception and empathy simultaneously. The EEG data were subject to representational similarity analyses (RSAs) to disentangle dynamic neural representations of perceived race and pain, similar to previous studies (e.g. Hall-McMaster et al. 2019). The results of RSA allowed us to clarify whether brain responses to perceived race or pain serve as a neural mediator of the associations between EG/RI and racial outgroup derogation. As the brain–behavior association observed in brain imaging research can be strongly inflated in studies with small sample sizes (Marek et al. 2022), we also estimated the necessity of the large sample size of our EEG data for relating individual differences in brain function to variations in the complex cultural trait (i.e. interdependence). Together, the findings of the current work revealed multiple components of interdependent self-construals that are associated with punishment decision-making to racial outgroups during intergroup conflicts in opposite directions.

## Materials and methods

### Study 1

#### Participants

In study 1, we recruited undergraduate and graduate Chinese students as paid volunteers (*n* = 2,297; 1,181 males; 1,041 females; mean age ± SD = 19.5 ± 2.6 years; 75 participants did not provide their gender information; 218 participants did not provide their age information). No participants reported psychological illness or taking any drugs when being tested. All participants provided written informed consent after the experimental procedure had been fully explained. The participants completed the Self-Construal Scale (Singelis 1994) to estimate their cultural orientations. This questionnaire consists of 24 items, with 12 items for the estimation of independence and 12 items for the estimation of interdependence that require ratings on a 7-point Likert scale (1 = strongly disagree, 7 = strongly agree). These participants were also tested in other experiments and the results were published in our previous research (Luo, Li, et al. 2015; Luo, Ma, et al. 2015).

### Data analyses

Initially, we conducted confirmatory factor analysis (CFA) to test whether the original 2-factor (interdependent and independent) model would be a sufficient fit to the data. The 2 latent variables (i.e. interdependent and independent) were calculated by the original methods described in previous literature (Singelis 1994) and were examined by CFA. As the CFA results did not indicate adequate fit of the 2-factor model, we did an exploratory factor analysis (EFA) to reveal the factor structure of the self-construal scale of our current sample. The details of CFA and EFA are provided in the supplementary materials (Supplementary Fig. S1, Supplementary Table S1).

### Study 2

#### Participants

The EEG data in study 2 was collected as part of the large-sample EEG project. All participants provided written informed consent after the experimental procedure had been fully explained. Participants were reminded of their right to withdraw at any time during the study. One thousand and six Chinese participants were recruited in study 2 (143 males, 814 females; 49 participants did not respond to the questionnaire but performed the EEG test; mean age ± SD = 20.5 ± 1.0 years). No participants reported psychological illness or taking any drugs when being tested. Exclusion criteria included limited analyzed trials (trial number < 10) due to artifacts or technical issues that occurred during EEG data collection (91 participants were excluded) and incomplete questionnaires provided by participants (239 participants were excluded). This resulted in *n* = 676 in the final example (120 males; 556 females; mean age ± SD = 20.6 ± 1.0 years).

After data collection, we conducted a power analysis with a sample size calculator for structural equation modeling (SEM) (Soper 2017). This analysis helps us to examine whether we had an appropriate sample size to detect the associations between the variables. This sample size calculator is based on an algorithm by Westland (2010), which determines the minimum sample size to detect reliable associations between latent variables. We assumed a small to medium effect size of 0.15 here, similar to the previous studies (Haraldsen et al. 2019; Lau et al. 2019). With a maximum of 2 latent and 11 observed variables, 679 participants were required to detect the effect with the desired power of 0.95. Both studies were approved by the local Research Ethics Committee in China.

### Stimuli and procedure

The stimuli in the EEG experiment consisted of 64 color photos of 16 Asian (8 females) and 16 White faces (8 females) adopted from our previous study (Sheng and Han 2012). Each model contributed 1 photograph of a pain expression and 1 photograph of a neutral expression. Emotional intensity, attractiveness, and luminance of Asian and White faces were matched (Sheng and Han 2012). Each face subtended a visual angle of 3.8° × 4.7° at a viewing distance of 60 cm in the EEG experiments. Each trial consisted of a face stimulus with a duration of 200 ms, which was followed by a fixation cross with a duration varying randomly between 800 and 1,400 ms. There were 4 blocks of 128 trials, with each photograph presented twice in a random order. Participants performed race judgment (Asian vs. White) on each photo by pressing 1 of 2 buttons using the right index or middle finger. The relationship between response buttons and Asian/White faces was also counterbalanced across different blocks of trials. Participants were encouraged to respond as fast and accurately as possible. Before the EEG recording, participants completed the Self-Construal Scale (Singelis 1994) to estimate their cultural orientations (i.e. interdependence and independence).

To quantify punishment decisions related to racial outgroup members in an intergroup conflict situation, before EEG recording, we asked participants to complete a behavioral test adopted from our previous study (Luo, Li, et al. 2015). Participants were first informed that, in an experiment, an Asian and a White student would receive an electric shock with a default intensity of 2.1 mA that would induce a moderate painful feeling. The White student was asked to modify the intensity of electric shocks to the Asian student between 0.8 mA, which induces a nonpainful sensory feeling, and 3.4 mA, which induces an intolerant painful feeling. The White student decided to apply a 2.8-mA shock (the high conflict condition) or a 1.5-mA shock (the low conflict condition) to the Asian student. Thereafter, our participants were asked to decide an intensity of an electric shock that would be applied to the White student in either the high conflict or low conflict conditions. Punishment decision-making was quantified as the difference in shock intensities in the high conflict versus low conflict conditions to control the effects of other processes that are involved in decision-making but are not specific to punishment. The punishment decisions on other-race targets in a similar context were associated with specific neural activations in the nucleus accumbens (Luo, Li, et al. 2015). To further validate the uniqueness of outgroup punishment decisions measured in our behavioral test, we performed an independent study (*n* = 44) in which participants were asked to decide punishments of a White target and an Asian target who were involved in conflict with an Asian (see Supplementary Information for details). The results showed that participants punished other-race (vs. same-race) targets more harshly when individual differences in general tendencies of punishing others were controlled (see Supplementary Information for details, Supplementary Fig. S2). Together, these results suggest that our measures of punishment decisions are different between ingroup and outgroup targets in a similar conflict context.

### E‌EG data acquisition and analysis

#### E‌EG data acquisition and preprocessing

A NeuroScan system (CURRY 7, Compumedics Neuroscan) was used for EEG recording and analysis. The EEG signal was continuously recorded from 32 scalp electrodes and was rereferenced to the average of the left and right mastoid electrodes offline. Impedances of individual electrodes were kept <5 kΩ. Eye blinks and vertical eye movements were recorded using electrodes located above and below the left eye. The horizontal electro-oculogram was recorded from electrodes placed 1.5-cm lateral to the left and right external canthi. The EEG signal was digitized at a sampling rate of 1,000 Hz and was subjected to an online band-pass filter of 0.01–400 Hz. EEG data were filtered with a low-pass filter at 30 Hz offline; 0.01 HZ was used as the high-pass filter in reference to the previous study that conducted RSA analyses of EEG data (Hall-McMaster et al. 2019). Artifacts related to eye movement or eye blinks were removed using the covariance analysis tool implemented in CURRY 7 (Semlitsch et al. 1986). The EEG data were downsampled to 100 Hz to reduce the processing time and increase the signal to noise ratio (Grootswagers et al. 2017; Proklova et al. 2019). The EEG data were epoched in accordance with stimulus trigger codes from −200 to 600 ms relative to stimulus onset. Trials contaminated by eye movements and muscle potentials exceeding ±100 μV at any electrode were excluded from the average. The EEG data were then epoched into 8 different conditions; i.e. 2 (race: Asian vs. White) × 2 (expression: pain vs. neutral) × 2 (gender: male vs. female).

#### RSAs of EEG data

We used RSA methods (Dobs et al. 2019; Hall-McMaster et al. 2019) in the EEG sample to disentangle the temporal profile of the neural representation of race, pain, and gender dimensions in face processing. We created the model representational dissimilarity matrix (model RDM) which were 8 × 8 matrices, where 1 corresponded to a between-category stimulus and 0 corresponded to a within-category stimulus comparison. This procedure resulted in 3-face model RDM corresponding to the race, pain, and gender dimensions of the stimuli.

For the computation of neural dissimilarity at each time points, we used the Mahalanobis distances (MDs) between conditions. This measure has been reported in the previous EEG study (Hall-McMaster et al. 2019) as an indicator of neural dissimilarity between conditions. This measure (i.e., MD) explicitly take covariance into account which make it well suited to EEG data in which channel values are highly correlated. Similar to the previous studies, the MDs between 2 conditions were computed based on the topography differences between 2 conditions and the channel covariance matrix. The MD between condition A and B is formally computed as follows: 


\begin{align*}&{\mathrm{MD}}_{\mathrm{AB}}=\!\sqrt{{\left(\mathrm{Pattern}\ \mathrm{A}-\mathrm{Pattern}\ \mathrm{B}\right)}^{\mathrm{T}}\!\times\! {\mathrm{Cov}}^{-1}\left(\mathrm{Pattern}\ \mathrm{A}-\mathrm{Pattern}\ \mathrm{B}\right)}\end{align*}

where Pattern A − Pattern B refers to the difference between topographies, T is the transpose, and Cov^−1^ is the inverse of the channel covariance matrix. The topography is a vector of 30 channel values (the values recorded from the left and right mastoid electrodes were deleted), which has been averaged over trials in each condition at each time point. The channel covariance matrix was measured via the trials × channels matrix. Before its computation, all trials in the trials × channel matrix were subtracted from the mean responses within that condition. The covariance calculation also used a shrinkage estimator (Ledoit and Wolf 2004), which has the effect of downweighting noisy covariance estimates. After the calculation of MDs between all pairs of conditions, we constructed an 8 × 8 EEG data RDMs as the indicator of neural dissimilarity matrix.

Next, the neural RDM and model RDMs were *z*-scored and were transformed into a vector. The neural and model distance vectors were then entered into a multiple regression analysis that was conducted at each time point. The regressions were performed separately for each participant, and the neural dissimilarity (i.e. the EEG data) at each time point was modeled as a linear combination of race, pain, and gender model dissimilarity. This produced 3 beta weights for each participant, which represented the unique variance contributed from the corresponding model (i.e. race, pain, and gender). The time courses of the resulting beta weights for each predictor were tested against 0 via nonparametric cluster-based permutation test (Maris and Oostenveld 2007; Hall-McMaster et al. 2019). Specifically, adjacent time points exceeding a predefined threshold (*P* < 0.05, 2-tailed) were grouped into 1 or multiple clusters. The summed cluster *t*-values were compared against a permutation distribution that was generated by randomly reassigning condition membership for each participant (10,000 iterations) and by computing the maximum cluster mass on each iteration. For all analyses, the cluster-based permutation tests were performed from 0 to 600 ms after the onset of faces.

#### SEM analyses

We first sought to crossvalidate the multifactorial structure of interdependence derived in study 1 using CFA. As compared to the model in which interdependence served as a global factor, the multifactorial construct of interdependence produced a better fit for Chinese samples (CFI_diff_ = 0.103); we then tested the relationship between the subcomponents of interdependence and race-related punishment decisions. To this end, we performed SEM analyses which have the strength of considering the interrelationships among different variables.

The outgroup punishment decisions during intergroup conflicts were measured by the difference of punishment decisions toward outgroup in high versus low conflict situations (see Stimuli and procedure for details). To examine the associations between subcomponents of interdependence with outgroup punishment behavior, we included 2 subcomponents of interdependence (i.e. EG and RI) as the latent variables in the model to predict the outgroup punishment decisions. The calculation of latent variables was implemented in the lavaan package (Rosseel 2012) in R v3.5.1. To further visualize the results (Fig. 2), we also extracted the factor scores of EG and RI from the SEM model using the function predict() implemented in the lavaan package (Rosseel 2012) in R v3.5.1. The factor scores of EG (or RI) were then standardized and regressed out the influence of RI (or EG). Besides, we also fitted an alternative model in which interdependence alone served as latent variable. We also examined the influence of EG/RI on the neural representation of race and pain. The neural indicators of representation of race and pain were calculated by the corresponding beta weights calculated in the RSA analysis. We chose the mean beta values between 120 and 160 ms, as the peak of the beta weights for both race and pain RDMs were 140 ms. Similarly, we fitted an alternative model with interdependence alone serving as latent variable. The estimation methods and fitting indices for the SEM and CFA analyses were similar to that of study 1. All analyses were conducted using R v3.5.1 (www.r-project.org). The EFA was conducted using the package psych (Revelle 2018), and CFA and SEM were conducted using the package lavaan (Rosseel 2012).

#### Mediation analysis

We conducted mediation analyses to examine the neural process behind the association between the subcomponents of interdependence (i.e. EG and RI) and the racial outgroup punishment decisions using the linear regression model implemented in the PORCESS for SPSS macro (model 4) (Hayes 2017). The factor scores of EG (RI) were calculated, standardized, regressed out the influence of RI (EG), and were then entered as independent variables here. The neural representation of race was placed as the mediator here. The significance of the mediation effect was estimated by using the bias-corrected bootstrapping method of PROCESS macros (*k* = 10,000 bootstrap repetitions, α = 95% confidence interval). The significance of all other effects (relationship between initial predictor and mediator, between mediator and outcome, and direct effect and total effect) were estimated with linear regression models implemented in the PROCESS macro. *P* values were then obtained for these effects, and the significance of the indirect effect was determined by whether or not 0 was included in the bootstrap confidence interval.

### Sampling variability analysis

Finally, we examined the effect of sample sizes on the distributions of correlations between EG/RI and outgroup punishment decision/neural representations of race in study 2. We randomly selected participants with replacements from the full behavioral sample (*n* = 676) at logarithmically spaced sample sizes (25, 29, 35, 42, 50, 59, 71, 84, 100, 119, 142, 169, 200, 239, 284, 338, 401, 478, 568, and 676; 20 intervals in total). At each interval, we randomly sampled participants for 1,000 times and calculated the correlations between EG/RI scores and outgroup punishment decision-/race-related beta weight obtained in the RSA analysis, resulting in 20,000 correlations for each association. For each association, we quantified sampling variability at each sampling interval as the range of correlations, with the 99% and 95% confidence intervals of correlations observed through this resampling procedure.

## Results

### Decomposition of interdependence

In study 1, we first tested whether self-construals in Chinese can be appropriately captured by the 2 global factors of interdependence and independence. To this end, we conducted a CFA of rating scores of the Singelis self-construal scale (24 items) obtained from a Chinese sample (*n* = 2,297). Specifically, we examined whether the rating scores fit the 2-factor model that consists of interdependence and independence as 2 latent variables. The results, however, showed that the 2-factor model provided poor fit to the data in this Chinese sample, χ^2^ = 3991.333, df = 251, *P* < 0.001, RMSEA = 0.081 (0.078–0.083), CFI = 0.746, SRMR = 0.070, suggesting inadequate interpretations of Chinese self-construals using the 2 global factors of interdependence and independence.

**Fig. 1:**
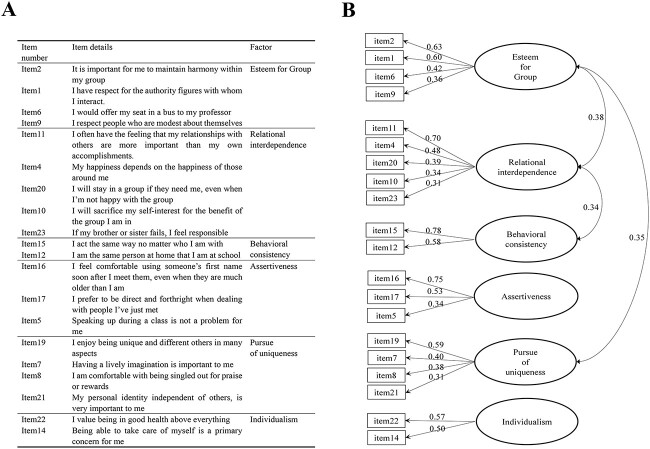
Results of the EFA. A) Items in self-construal scale and their corresponding factors in the 6-factor model. B) The 6-factor model of self-construals. The unidirectional arrows indicate the factor loadings of each item. The bidirectional arrows indicate intercorrelations between factors. EG (i.e., Esteem for Group) and RI (i.e., Relational interdependence) are from the original interdependence scale, whereas assertiveness, pursue of uniqueness, individualism, and behavioral consistency are from the original independence scale.

We also examined whether models with more than 2 factors provide a better fit to the data by conducting an EFA. The analysis identified 6 factors (Fig. 1, see Supplementary results section for details) and disclosed 2 subdimensions of interdependence, including EG (items such as “It is important for me to maintain harmony within my group”) and RI (items such as “I often have the feeling that my relationships with others are more important than my own accomplishments”). These subdimensions of self-construals derived from our Chinese sample was similar to the one constructed from an American sample (Hardin et al. 2004).

### Two subcomponents of interdependence—crossvalidation

Given that study 1 disclosed 2 subcomponents of interdependence using the EFA, in study 2, we sought to crossvalidate the multifaceted nature of interdependence by collecting self-construal rating scores from an independent Chinese sample. We conducted a CFA of participants’ self-construal rating scores. The results showed that the EG and RI factors obtained in the EFA in study 1 reasonably fitted the data in study 2; χ^2^ = 114.917, df = 26, *P* < 0.001, RMSEA = 0.071 (0.058–0.085), CFI = 0.947, SRMR = 0.052. We also tested the model in which the interdependence served as a global factor, but results showed poor fit compared with the multifaceted interdependence model; χ^2^ = 471.070, df = 54, *P* < 0.001, RMSEA = 0.107 (0.098–0.116), CFI = 0.844, SRMR = 0.078, CFI_diff_ = 0.103. Taken together, the results from 2 independent samples in studies 1 and 2 provided crossvalidation of the 2 subcomponents of interdependence (i.e. EG and RI) in Chinese.

### Opposite relationships between EG/RI and race-related punishment decisions

**Fig. 2:**
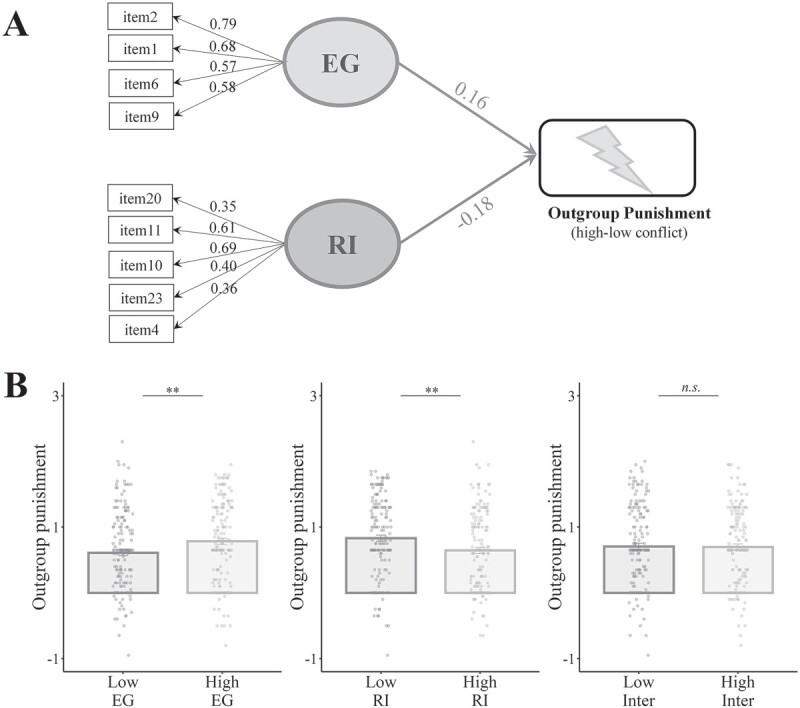
Associations between EG/RI and race-related punishment decisions. A) SEM models relating EG and RI to outgroup punishment decisions. Significant regression parameters are shown in solid lines. All parameter estimates shown are fully standardized. B) Bar plots illustrating the outgroup punishment decisions for individuals endorsed with low and high EG/RI or interdependence scores.

Next, we tested whether EG and RI are associated with punishment decisions related to racial outgroup individuals in opposite directions. We asked the participants in study 2 to perform a punishment decision task (Luo, Li, et al. 2015), which required the selection of intensities of painful electric shocks that were supposed to be applied to a racial outgroup target who had been engaged in either low or high conflict with a racial ingroup individual (see Stimuli and procedure for details). The results showed that the participants applied stronger painful shocks to the target in the high than low conflict conditions (*t*(675) = 31.67, *P* < 0.001). We estimated potential relationships between EG/RI and outgroup punishment decisions by conducting a SEM analysis. This analysis included EG and RI as 2 latent variables and punishment decisions in high (vs. low) conflict conditions as a dependent variable. The results indicated good fit; χ^2^ = 130.261, df = 33, *P* < 0.001, RMSEA = 0.066 (0.054–0.078), CFI = 0.942, SRMR = 0.050, Fig. 2A. Importantly, EG was positively associated with the intensity of electric shocks for punishment toward the racial outgroup target (*Z* = 2.75, β = 0.155, *P* = 0.006), whereas RI was negatively associated with the decision (*Z* = −3.36, β = −0.185, *P* = 0.001, Fig. 2A).

To test whether EG and RI predicted the outgroup punishment decisions irrespective of participants’ gender, we conducted another SEM in which gender was included as a covariate. Similarly, the results showed that EG was positively associated with the punishment decisions toward the racial outgroup target (*Z* = 2.77, β = 0.188, *P* = 0.006), whereas RI was associated with the decisions in the opposite direction (*Z* = −3.37, β = −0.223, *P* = 0.001). We also fitted an alternative model in which interdependence served as the latent variable to associate with the punishment decisions toward the outgroup. The model did not fit the data well; χ^2^ = 499.59, df = 65, *P* < 0.001, RMSEA = 0.100 (0.091–0.108), CFI = 0.839, SRMR = 0.076, Supplementary Fig. S3. The rating score of interdependence as a global factor failed to associate with the punishment decisions (*Z* = −0.68, β = −0.027, *P* = 0.496, Supplementary Fig. S3).

To illustrate and visualize individual differences in outgroup punishment decisions, we calculated factor scores of EG and RI. The factor scores of EG (RI) were then standardized and regressed out the influence of RI (EG). We selected participants with low (bottom 25th percentile participants) and high (top 25th percentile participants identified by this index) EG/RI factor scores. We compared the outgroup punishment decisions between these 2 groups and confirmed significant larger outgroup punishment decisions for the high (vs. low) EG groups (*P* = 0.004, Wilcoxon rank-sum test, Fig. 2B), whereas we confirmed significant smaller outgroup punishment decisions for the high (vs. low) RI groups (*P* = 0.003, Wilcoxon rank-sum test, Fig. 2B). Similarly, we also sorted participants into high and low interdependence score groups but failed to find significant differences in outgroup punishment decisions between the 2 groups (*P* = 0.982, Wilcoxon rank-sum test, Fig. 2B). Together, these results suggest that the subcomponents of interdependence are better than interdependence as a global factor for the interpretation of individual differences in outgroup punishment decisions.

### Opposite relationships between EG/RI and neural representations of race

**Fig. 3:**
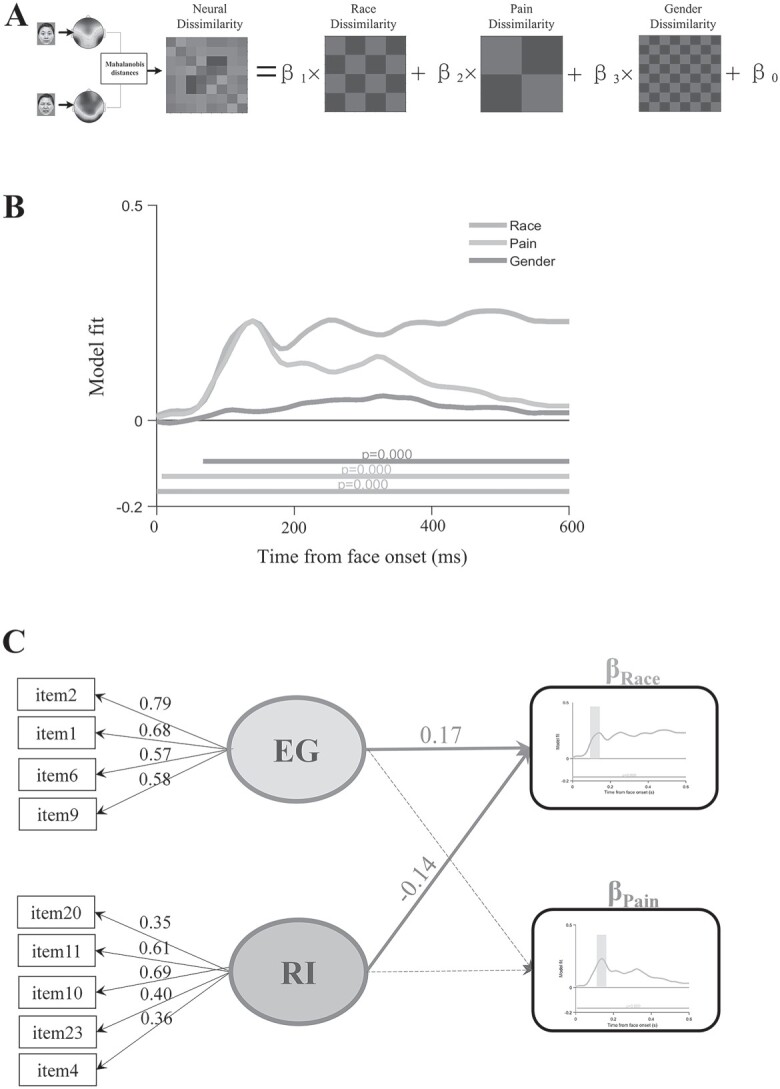
Results of the RSAs. A) Illustration of the RSA model. At each time point, the neural dissimilarity matrix between conditions was constructed by calculating the MD for all pairs of conditions, which captures the multivariate distance between topographies. This neural dissimilarity matrix was then modeled as a linear combination of models based on race, pain, and gender dissimilarity (1 corresponding to between and 0 corresponding to within category, respectively). This analysis produced one beta estimate time course for each dimension at each time point. B) RSA results using MD. The time courses of regression weights show the contributions of race, pain, and gender dissimilarity to neural dissimilarity for the large sample EEG participants (*n* = 676). Lines below plots indicated significant times using a cluster-based permutation test (cluster-defining threshold *P* < 0.05). C) SEM models relating EG and RI to early neural representations of race and pain. Significant regression parameters are shown in solid lines. Insignificant regression parameters are shown in dashed lines.

To examine possible neural mediators of the association between cultural traits and race-related punishment decisions, in study 2, we recorded EEG signals from participants while they responded to the racial identities of Asian and White faces with painful or neutral expressions (Sheng and Han 2012; see Stimuli and procedure for details). To test whether EG and RI place influence on neural representations of race or pain, we dissociated the neural representations of race, pain, and gender by conducting an RSA. Specifically, the neural dissimilarity of EEG signals at each time point was modeled as a linear combination of theoretical models capturing the dissimilarity on race, pain, and gender dimensions for stimulus pairs (i.e. 1 for between and 0 for within category stimulus pairs, Fig. 3A). This analysis produced a beta estimate time course for each dimension. A larger beta estimate represents a greater contribution of a specific dimension (i.e. race, pain, or gender) to neural dissimilarity at each time point. Similar to previous research (Hall-McMaster et al. 2019), the neural dissimilarity matrices were assessed using the Mahalanobis distances (MDs) of EEG signals between 2 conditions, representing the multivariate distance between topographies (Fig. 3A; see RSAs of EEG data in Materials and methods section for details). Consistent with previous findings (Sheng and Han 2012; Zhou et al. 2020), our results revealed similar early neural representations of race and pain (peak at 140 ms, both cluster *P* < 0.001, Fig. 3B), though the neural representation of race was larger compared to pain after 190 ms (cluster *P* < 0.001). The extraction of gender information peaked later compared to those of race and pain (peak around 300 ms, cluster *P* < 0.001, Fig. 3B). Overall, the results suggest early extraction of race and pain information during face perception.

Next, we calculated the averaged beta weights for representation dissimilarity matrices of race and pain at 120–160 ms as indices of early neural representations of race and pain (the time window was chosen as both race and pain neural representation peaked at 140 ms). The beta weights were then subjected to SEM analysis to examine potential relationships between the neural indicators of race and pain and the latent variables (i.e. EG and RI) derived from the multifactor model of self-construals (Fig. 3C). The model fitted the data well; χ2 = 137.843, df = 40, *P* < 0.001, RMSEA = 0.060 (0.049–0.071), CFI = 0.942, SRMR = 0.047. Importantly, EG was positively associated with the early neural representation of race (*Z* = 3.408, β = 0.174, *P* = 0.001), whereas RI showed a negative association (*Z* = −2.516, β = −0.142, *P* = 0.012), suggesting opposite relationships of neural representations of race with EG and RI. However, EG (*Z* = −1.203, β = −0.063, *P* = 0.229) and RI (*Z* = 0.498, β = 0.029, *P* = 0.618) were not associated with early neural representation of pain. We also added the averaged beta weights for representation dissimilarity matrices of gender at 120–160 ms into the SEM, but neither the EG (*Z* = 1.413, β = 0.080, *P* = 0.158) nor RI (*Z* = −0.402, β = −0.023, *P* = 0.687) was associated with neural representations of gender, while the association between EG/RI and the neural representation of race remained significant (EG/RI: *Z* = 3.405/−2.515, β = 0.173/−0.142, *P* = 0.001/0.012). These results reinforce the association between race-related processing and the subcomponent of interdependence and suggest that these associations were not influenced by gender-related processing of faces.

Similarly, to test whether the associations between EG/RI and neural representations of race were not influenced by participants’ gender, we conducted another SEM analysis by including gender as a covariate. After controlling the influence of gender, EG still positively predicted the early neural representation of race (*Z* = 2.98, β = 0.151, *P* = 0.003), whereas RI negatively predicted the early neural representation of race (*Z* = −2.12, β = −0.119, *P* = 0.034). Neither EG (*Z* = −1.56, β = −0.083, *P* = 0.118) nor RI (*Z* = 0.84, β = 0.049, *P* = 0.399) was associated with the early neural representation of pain in this model. We also tested an alternative model which only included the global factors of interdependence, but the model did not fit the data well; χ^2^ = 495.220, df = 76, *P* < 0.001, RMSEA = 0.090 (0.083–0.098), CFI = 0.846, SRMR = 0.070, Supplementary Fig. S4. Moreover, the interdependence as a global factor was not associated with neural representations of either race (*Z* = 1.619, β = 0.064, *P* = 0.105) or pain (*Z* = −0.983, β = −0.042, *P* = 0.325). These results suggest that the global factor of interdependence is not sufficient to capture the individual differences in the neural representations of race during face perception. The subcomponents of interdependence, however, well predict neural representations of race.

### Neural representations of race mediate associations between EG/RI and punishment decisions

We further tested possible indirect paths from EG/RI to outgroup punishment decisions during intergroup conflicts via early neural representations of race. We first validated the relationship between the neural representation of race and outgroup punishment decisions. To this end, we conducted a regression analysis in which the neural representations of race, pain, and gender (i.e. all neural indicators derived through the RSA approach) served as independent variables and the outgroup punishment decisions in the high (vs. low) conflict conditions served as a dependent variable. The results showed that the neural representation of race was positively related to the outgroup punishment decisions (β = 0.122, *t* = 3.144, *P* = 0.002), whereas the other 2 neural indicators of pain and gender were not associated with participants’ punishment decisions (pain/gender: β = 0.036/−0.044, *t* = 0.947/−1.138, *P* = 0.344/0.256).

**Fig. 4:**
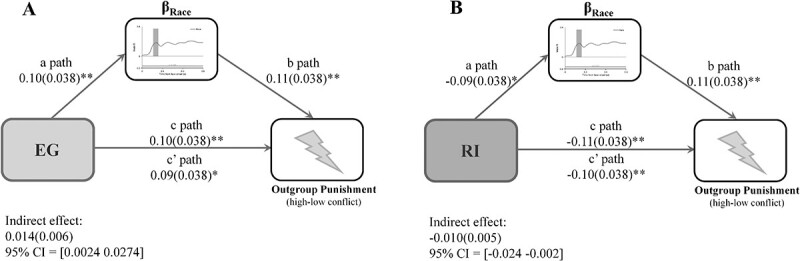
Illustration of the results of mediation analyses. The beta weight for race RDM at the early time window (120–160 ms) mediates A) the positive relationship between EG and racial outgroup punishment behavior and B) the negative relationship between RI and racial outgroup punishment behavior.

We then conducted mediation analyses that included the orthogonalized factor scores of EG or RI as independent variables, beta values for representation dissimilarity matrices of race at 120–160 ms as a mediating variable, and punishment decision as an outcome variable. The results first revealed that early neural representations of race were positively associated with EG (*t* = 2.73, β = 0.10, *P* = 0.0065) but were negatively associated with RI (*t* = −2.28, β = −0.09, *P* = 0.02). Moreover, enhanced early neural representations of race predicted greater intensity of electric shocks for punishing other-race targets in the high (vs. low) conflict conditions after controlling the influence of EG and RI on punishment decision-making (*t* = 2.83 and 2.85, β = 0.11 and 0.11, *P* = 0.0048 and 0.0045). Most importantly, early neural representations of race mediated both the positive relationship between EG and intensity of electric shocks assigned to other-race targets (indirect effect = 0.0114 bootstrap 10,000 repetitions, 95% confidence interval = 0.0024–0.0274, Fig. 4A) and the negative relationship between RI and the intensity of electric shocks assigned to other-race targets (indirect effect = −0.0091 bootstrap 10,000 repetitions, 95% confidence interval = −0.0241 to −0.0016, Fig. 4B). The results suggest the early neural representation of race as a potential intermediate mechanism underlying the associations between EG/RI and racial outgroup punishment decisions.

Previous research showed that empathy for others’ pain motivates reactive punishment (Pfattheicher et al. 2019) and that neural responses to ingroup suffering were related to outgroup punishment decisions (Han et al. 2020). Therefore, although the general neural representation of pain was not associated with the outgroup punishment decisions (Fig. 3), we further tested if the pain-related processing for White or Asian faces was associated with the outgroup punishment. To this end, we conducted separate RSA analyses for White and Asian faces, similar to previous research (Dobs et al. 2019). We found that the neural representation of pain was significantly greater for Asian faces than for White faces (Supplementary Fig. S5), and this was consistent with our previous research using univariate analyses showing the racial ingroup bias in empathy for pain (Sheng and Han 2012). We then tested potential associations of the neural representation of pain for Asian (or White) faces with the outgroup punishment decisions in the high (vs. low) conflict situations, respectively. The results, however, did not show significant correlation (*P*s > 0.090, see Supplementary Table S2).

### Sample variability of the associations of EG/RI with outgroup punishment decisions and neural representations of race

**Fig. 5:**
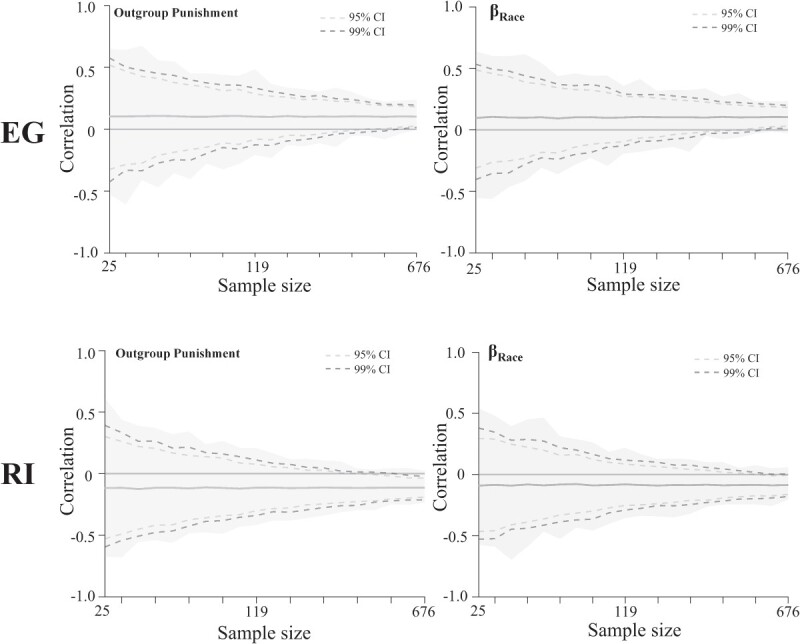
Illustration of sampling variability of the associations of EG/RI scores with outgroup punishment decisions and neural representations of race. The correlation coefficients between EG/RI scores and outgroup punishment decision (left panels) and the correlation coefficients between EG/RI scores and the race-related beta weight (right panels) are shown. Solid lines show the mean across 1,000 resamples for the corresponding associations. Shading represents the minimum to maximum correlation range across subsamples for a given sample size. Dashed lines represent 95% and 99% confidence intervals.

Finally, we assessed the effect of sample sizes on the distributions of correlations between EG/RI scores and outgroup punishment decisions as well as between EG/RI scores and neural representations of race in study 2. We randomly selected participants with replacements from the full behavioral sample (*n* = 676) at 20 intervals (*n* = 25–676) and calculated the associations between EG/RI scores and outgroup punishment decisions/race-related beta weight obtained in the RSA analysis (see Sampling variability analysis in Materials and methods section for details). In Fig. 5, we charted the sampling variability of correlation coefficients of these associations as a function of sample size. The sampling variability of 99% and confidence intervals at each sampling interval are shown in Supplementary Tables S3 and S4. The results indicate that the associations of EG/RI scores with both outgroup punishment decisions and neural representations of race can be inflated by chance in small samples. For example, at *n* = 25, the 99% confidence interval for the association between EG and outgroup punishment was [−0.43, 0.58], while the actual mean coefficient was 0.10. At *n* = 568, however, the 99% confidence interval for the association between EG and outgroup punishment was [0.00, 0.20], while the actual mean coefficient was 0.10. Together, the results of these analyses suggest the necessity of the large sample size of our EEG data for relating individual differences in punishment decision-making and in race-related brain function to variations in complex cultural traits such as the subcomponents of interdependence.

## Discussion

The present study investigated the relationships between interdependent self-construals and outgroup punishment during intergroup conflicts by collecting self-construal scores, outgroup punishment decisions, and EEG data. We conducted multiple analyses to identify and crossvalidate the subcomponents of interdependence (i.e. EG and RI), to examine relationships between EG/RI and punishment decisions on racial outgroup targets, and to explore relevant potential neural mediators. Our findings highlight opposite relationships of EG and RI with outgroup punishment behavior in the context of intergroup conflicts and unravel potential neural mediators.

The 2 global factors of self-construals (i.e. interdependence and independence) have been used widely in behavioral and brain imaging studies to interpret cultural differences in and cultural influences on cognition, emotion, and behavior (Triandis 1989; Markus and Kitayama 1991; Cross et al. 2011; Han et al. 2013). These cultural traits have also been linked to brain structures, such as gray matter volumes in specific brain regions, including the medial prefrontal and orbital cortices (Kitayama and Salvador 2017; Wang et al. 2017), and functional connectivity of brain activities during resting states (Li et al. 2018). However, the 2 global factors of interdependence and independence do not explain well all social behaviors such as the case of outgroup punishments during intergroup conflicts. It seems that complicated social behaviors cannot be simply mapped to the 2 global dimensions of self-construals due to the multidimensional nature of self-construals (Oyserman et al. 2002; Hardin et al. 2004; Hardin 2006).

Indeed, the findings of our current work support the latter proposition by showing evidence that, relative to the model in which interdependence serves as the global factor, a multifaceted interdependence model provides a better fit for the empirical estimation of self-construals. Specifically, our results revealed EG and RI as 2 subcomponents of the global factor of interdependence. Together with the results of previous research that tested an American sample (Hardin et al. 2004), our findings indicate the presence of 2 subcomponents of interdependence in both collectivistic and individualistic societies. The multifactor model also revealed subcomponents of self-construals which correspond to the global factor of independence, which was similar to previous research on American samples (Hardin et al. 2004). These findings implicate the necessity to analyze potential multifactorial structures of other cultural traits for a better understanding of the nature of personality that characterizes individuals in different societies.

More importantly, our results provide evidence that 2 subcomponents of interdependence are oppositely associated with outgroup punishment decision-making in the context of intergroup conflicts. Specifically, we showed evidence that individuals with greater EG gave stronger punishment toward the racial outgroup target, whereas individuals with greater RI gave weaker punishment. These findings of the opposite relationships between outgroup punishments and EG/RI provide new insights into our understanding of why the global factor of interdependence does not predict the individuals’ punishment decisions toward the outgroup. An important implication of these findings is that, if the subcomponents of interdependence drive social behavior in opposite directions, the global factor of interdependence has to be broken into subcomponents in order to better predict complex social behavior in future studies.

The opposite relationships between EG/RI and outgroup punishment behavior provide a possible interpretation of previous seemingly incongruent findings. For example, researchers have reported inconsistent findings suggesting that interdependence might be associated with either increased (Cheon et al. 2011; Gelfand et al. 2012; Duclos and Barasch 2014; Wang et al. 2015) or decreased (Gouveia 2011) outgroup deficits in empathy or outgroup aggression during intergroup conflicts when comparing individuals from Western and Asian societies or when comparing individuals primed with interdependence or independence. The current findings suggest that the 2 global factors of self-construals might be oversimplified in the prediction of social behavior as well as in its psychological and neural underpinnings. The fact that EG and RI are linked to punishment decision-making pertaining to outgroup in opposite directions suggests the presence of nuanced neurocognitive processes that support outgroup punishment behavior in different ways. EG and RI extracted from the 2 samples of our study map closely to the collective interdependence and RI proposed by the previous theoretical model (Cross et al. 2000). The items that construct EG emphasize the tendency to define the self in terms of group collectives and strengthen the hierarchical nature of social relationships. The items that construct RI focus on self-sacrifice to maintain a harmonious relationship. Previous studies suggested that the pursuit of social hierarchy is related to less empathy for the pain to outgroup compared to ingroup in Asian samples (Cheon et al. 2011) and has been also associated with less empathy and stronger schadenfreude toward low-status people in Western samples (Hudson et al. 2019). The hierarchical cultural perspective assumes that social harmony is attained if everyone stays within his or her boundaries (Nisbet et al. 2001), and social norms like this may promote ingroup–outgroup boundary, which gives rise to increased outgroup punishment (Markus and Kitayama 2010). On the other hand, RI assigns high importance to social contact and harmony and promotes egalitarian views of social relationships (Gouveia et al. 2008; Gouveia 2011), cooperation (Yamagishi et al. 1998), and conflict-reducing behavior (Forbes et al. 2011), which may further lead to the decrease of outgroup punishment behavior in the context of intergroup conflicts.

Our current findings unraveled a potential neural mediator of the relationships between EG/RI and race-related punishment decision-making. The results of our RSA revealed the neural representation of race at 100–200 ms after face onset, similar to previous findings (Ito and Urland 2003; Zhou et al. 2020; Zhang and Han 2021). More interestingly, we showed that the early neural representation of race mediates the associations of both EG and RI with race-related punishment decision-making. Although the racial ingroup bias in empathic brain responses (e.g. enhanced neural responses to perceived painful (vs. neutral) expressions of same-race than other-race individuals) has been shown to predict ingroup help (Hein et al. 2010; Cheon et al. 2011) or outgroup punishment (Luo, Li, et al. 2015), it remains unclear whether neural representations of race or pain drives race-related decision-making separately. One possibility is that self-construals affect the perception of ingroup–outgroup distinction (Markus and Kitayama 2010), such as same-race/other-race categorization, which further leads to increased outgroup punishment. Consistent with this assumption, previous research found that people who showed high category salience (i.e. greater awareness of ingroup–outgroup distinction) also reported contacts with outgroups being more negative, suggesting their motivational and emotional biases when interacting with outgroups (Paolini et al. 2010). At the neural level, our recent study also discovered that enhanced neural coding of the other-race category predicted weaker altruistic intentions to help other-race individuals who suffer from painful stimulations (Zhou et al. 2020). This work, however, did not take cultural traits into consideration when examining the relationships between racial categorization and prosocial behavior. Alternatively, self-construals may generally influence attention to others’ emotional states (Cross et al. 2000), which further affects subsequent social decision-making.

The results of our mediation analyses in the current work showed that, although EG and RI were linked to outgroup punishment decisions in opposite directions, the early neural representations of perceived race may provide an intermediate mechanism that links both EG and RI to outgroup punishment behavior. The effect of cultural stereotypes (e.g. viewing Blacks and Whites as aggressive, violent, and dangerous) on shooting decision-making in response to armed and unarmed Black and White targets were mediated by neural activities related to racial differentiation (i.e. P200 and N200 components in ERP; Correll et al. 2006). These results highlight the functional role of neural representations of race that links race-related decision-making to both observers’ own cultural traits and cultural stereotypes about others. These findings provide empirical evidence that supports the proposition that self-construals may affect outgroup punishment by modulating the sensitivity to ingroup–outgroup distinctions (Markus and Kitayama 2010). The perception of ingroup–outgroup distinction is the precondition for intergroup conflicts. Our findings of positive relationships between neural representations of race and outgroup punishment during intergroup conflicts further reinforce the key role of category salience in intergroup conflicts (Turner et al. 1987; Han 2018; Zhou et al. 2020).

The findings of the current work provide further empirical evidence for the basis model of self-specificity (Northoff 2016). This model posits that self-specificity serves a basic internally based function, which is supported by the spontaneous activities in the default brain network, including the medial prefrontal cortex and posterior cingulate (Northoff et al. 2006; Qin and Northoff 2011), and provides a baseline for and acts through spatiotemporal schemata of specific tasks (Scalabrini et al. 2022). The neuro-ecological model of self further assumes that the neuro-social and neuro-ecological alignment of the brain to its respective environmental contexts leads to cultural dependences of both psychological and neuronal features of self (Scalabrini et al. 2021). According to these models, the subcomponents of interdependence (i.e. EG and RI) disentangled in our work served as the basic function of self-specificity or the baseline self that might be independent of temporal social task demands. The race-related processing as revealed in our EEG results, however, represents the higher-order self-related processing at the intergroup level (i.e. racial ingroup–outgroup distinction). The mediation model data in our study suggest that the interaction between the baseline self and higher-order self determined the racial outgroup punishment behaviors.

By showing evidence that EG and RI have opposite relationships with outgroup punishments, our work provides insights into potential interventions that sought to improve interracial relationships. Cultural psychologists propose that an individual may identify with and switch between multiple cultural systems (Hong et al. 2000). This idea led to investigations of how neural and behavioral responses are altered by modifying (or priming) individuals’ cultural traits. Previous research has shown that priming independence compared to interdependence self-construals decreased racial ingroup bias in empathic neural responses (Wang et al. 2015). Our current findings implicate that enhancing EG and RI using priming or other methods might influence outgroup punishments during intergroup conflicts in opposite directions. This can be tested in future research.

To maximize the power of our tests of the subcomponents of interdependence, we tested 2 large Chinese samples in studies 1 and 2. The results in study 1 were crossvalidated in study 2, indicating that the 2 subcomponents of interdependence can be repeatedly observed in different testing samples. Study 2 collected EEG and behavioral data from a sample that was much larger than those in previous EEG studies and was appropriate for the detection of associations between latent variables in the SEM. Besides, a large sample of EEG data reduced the possibility of inflated, irreproducible brain–behavior associations that were usually obtained in small sample research and reduced outliers’ influences on the results of our mediation analysis to a minimum degree. This is well illustrated in the results of our sampling variability analyses, which indicate the necessity of a large sample EEG data for establishing reliable associations between cultural traits and social decision-making as well as between cultural traits and brain activity supporting social decision-making. Despite the efforts to maximize the power of our tests, the current work tested only 1 cultural sample in a collectivistic society, and the samples were predominately based on undergraduate or graduate students. Previous research suggested that the multifactor model of self-construal was better than the original 2-factor model for both young adults and elderly people (Guo et al. 2008). Moreover, interdependence was divided into 2 subcomponents in both aging samples, similar to the current study. Future research should test whether EG and RI are oppositely associated with other-race punishment decision-making in other cultural samples as well as in different age groups. This is necessary to make a general conclusion regarding the relationships between EG/RI and outgroup punishment decision-making. Finally, the current work employed a race judgment task that guided participants’ attention to racial identity rather than emotional states of each face. This task measured spontaneous empathic responses but did not allow examination of neural activity involved in cognitive control during empathy. As previous work showed the pain-related processing was influenced by the level of attention (Sheng and Han 2012), future work may employ the pain judgment task to test whether the neural representations of pain are associated with outgroup punishment decisions when task demands shift attention to one’s emotional states.

## Conclusion

The current study disentangled EG and RI as the 2 subcomponents of interdependent self-construals by integrating factorial analyses of questionnaire measures from 2 large cohorts. Our results further revealed that EG and RI predicted outgroup punishment decision-making in opposite directions. In addition, we showed evidence for early neural representations of race as neural mediators of the associations between EG/RI and outgroup punishment in intergroup conflicts. Together, our findings identified the multifaceted nature of self-construals and advanced our understanding of the relationships between interdependent self-construals and racial outgroup punishment.

## Supplementary Material

Supporting_information_2023_04_final_submitted_bhad157

## Data Availability

The data that support the findings are available from the corresponding author, Yuqing Zhou with a reasonable request.
