# Supplementary material for: Neural representation of perceived race mediates the opposite relationship between subcomponents of self-construals and racial outgroup punishment
Source: Cereb Cortex. 2023 May 4;33(13):8759–72. doi: 10.1093/cercor/bhad157 (PMC10786092; doi:10.1093/cercor/bhad157)
Supplement: Supporting_information_2023_04_final_submitted_bhad157 [file supporting_information_2023_04_final_submitted_bhad157.docx]

**Supplementary Information**

**Neural representation of perceived race mediates the opposite relationship between subcomponents of self-construals and racial outgroup punishment**

**Yuqing Zhou^1,2^, Wenxin Li^3^, Tianyu Gao^4^, Xinyue Pan^3^, Shihui Han^3^**

**^1^CAS Key Laboratory of Behavioral Science, Institute of Psychology, Chinese Academy of Sciences, Beijing, 100101, China**

**^2^Department of Psychology, University of Chinese Academy of Sciences, Beijing, 100049, China**

**^3^School of Psychological and Cognitive Sciences, PKU-IDG/McGovern Institute for Brain Research, Beijing Key Laboratory of Behavior and Mental Health, Peking University, Beijing, China**

**^4^Department of Psychology, Faculty of Arts and Sciences, Beijing Normal University, Zhuhai, 519087, China**

Table S1-4

Figures S1-5

**Supplementary methods of Study 1**

The CFA were based on the weighted least squares means and variance adjusted (WLSMV) estimation method to account for the ordinal nature of the 7-point Likert self-construal items (Pagliaccio et al., 2016). The factor structure was evaluated based on goodness of fit measured by the χ2 statistic, the comparative fit index (CFI), Standardized Root Mean Square Residual (SRMR), and the root mean square error of approximation (RMSEA). Although a nonsignificant χ2 is the aim, a significant χ2 can be expected due to the large sample size of the current study (Bentler & Bonett, 1980). For the RMSEA and SRMR, values <0.08 indicate adequate fit and values < 0.05 indicate a good fit. For the CFI, values > 0.9 suggest a reasonable fit (Hu & Bentler, 1999).

EFA was performed on the 24 item self-construal scales. The EFA used the principal axis factoring (PAF) as the method of extraction, and subsequent oblique (promax) rotation. The parallel analysis (PA) was performed to determine the number of factors to retain before the EFA. PA generates a random set observations and variables equal to the number in the observed dataset. Using principal axis analysis, eigenvalues are extracted from the random data, and this is repeated for 1000 iterations. Eigenvalues from the 95th percentile of the random set are then compared to those from the observed data, and those with eigenvalues greater than the random data are retained. Items showed weak loadings (< 0.3) on all factor or cross-loading on multiple factor (> 0.3) were deleted from further analysis.

**Supplementary results of Study 1**

An initial parallel analysis was performed to estimate the number of factors and identified seven factors in our sample (Figure S1 for scree plots). Because one factor consists of only one item of self-construal scale (i.e., item 21), we decided to extract six factors for further analyses. The loadings of each item for the six factors are presented in Table S1. Three items (item 3, item 24, item 13) in the original scale were deleted because of weak factor loading (< 0.3), and one item (item 18) was deleted due to its cross-loading on two different factors (> 0.3 on both factors). The results of the 6-factor model revealed six subdimensions of self-construals in the Chinese sample. These included (1) EG (items such as “It is important for me to maintain harmony within my group”), (2) RI (items such as “I often have the feeling that my relationships with others are more important than my own accomplishments”), (3) assertiveness (items such as “I prefer to be direct and forthright when dealing with people I’ve just met”), (4) behavioral consistency (items such as “I act the same way no matter who I am with”), (5) pursue of uniqueness (items such as “I enjoy being unique and different from others in many aspects”) and (6) individualism (items such as “I value being in good health above everything”). The 6-factor model constructed from our Chinese sample was similar to the one constructed from an American sample (Hardin et al., 2004). The items pertaining to EG and RI come from the original interdependence scale whereas the items related to other subdimensions come from the original independence scale. These results indicate a better interpretation of self-construals in the Chinese sample by a 6-factor compared to a 2-factor model and disclose two subcomponents of interdependence (i.e., EG and RI).


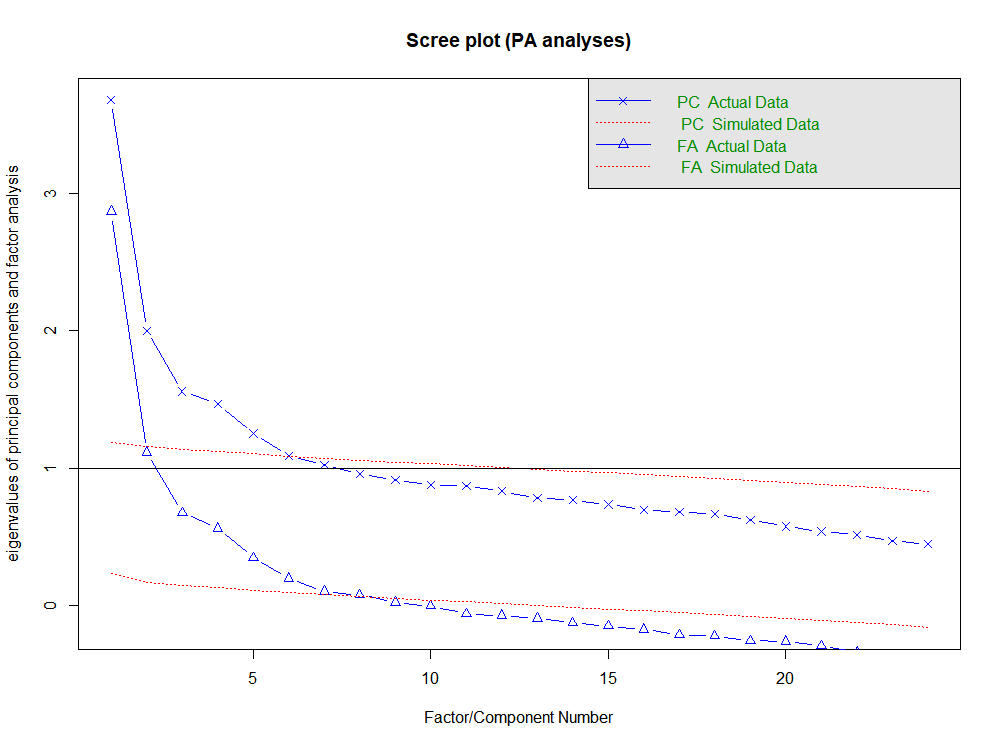


**Figure S1. Scree plot of Eigenvalues derived from the behavioral sample (N = 2297).** Shown are the results of a parallel analysis where eigenvalues from the behavioral sample data were compared to a Monte Carlo simulation of the data using permutations of the actual data. The plot shows seven factors above the 95th percentile line cutting the scree plot. Eigenvalues are on the y-axis.

**Table S1.** Factor loadings of each item in the self-construal scale of the EFA analysis

|  | Esteem for group | Relational interdependence | Assertiveness | Behavioral consistency | Uniqueness | Individualism |
| --- | --- | --- | --- | --- | --- | --- |
| item1 | **0.61** | -0.08 | 0.06 | -0.09 | 0.00 | -0.01 |
| item2 | **0.64** | 0.07 | 0.07 | -0.13 | -0.07 | 0.00 |
| *item3* | 0.01 | -0.05 | 0.06 | 0.07 | 0.08 | 0.21 |
| item4 | -0.04 | **0.46** | 0.09 | -0.11 | -0.04 | 0.04 |
| item5 | 0.00 | 0.00 | **0.36** | 0.02 | 0.19 | -0.02 |
| item6 | **0.39** | -0.03 | -0.02 | 0.04 | 0.14 | -0.11 |
| item7 | 0.14 | 0.08 | 0.01 | -0.08 | **0.40** | 0.02 |
| item8 | 0.11 | -0.04 | 0.06 | -0.10 | **0.36** | 0.08 |
| item9 | **0.44** | -0.02 | -0.06 | 0.09 | 0.08 | -0.04 |
| item10 | 0.28 | **0.39** | 0.06 | 0.16 | 0.01 | -0.21 |
| item11 | -0.07 | **0.67** | 0.12 | -0.02 | -0.08 | 0.10 |
| item12 | 0.00 | -0.04 | 0.07 | **0.59** | -0.12 | 0.04 |
| *item13* | 0.18 | 0.12 | -0.01 | 0.06 | -0.11 | 0.18 |
| item14 | -0.13 | 0.04 | -0.03 | 0.03 | 0.07 | **0.56** |
| item15 | -0.11 | 0.02 | 0.10 | **0.76** | -0.15 | 0.11 |
| item16 | 0.01 | -0.02 | **0.72** | 0.07 | 0.08 | -0.06 |
| item17 | 0.09 | 0.08 | **0.55** | 0.10 | 0.01 | 0.01 |
| *item18* | 0.37 | 0.38 | 0.13 | -0.02 | -0.05 | 0.01 |
| item19 | -0.27 | 0.15 | 0.15 | -0.07 | **0.57** | 0.00 |
| item20 | -0.09 | **0.41** | -0.08 | 0.10 | 0.16 | -0.14 |
| item21 | 0.08 | -0.13 | -0.04 | 0.04 | **0.33** | 0.16 |
| item22 | -0.01 | 0.10 | -0.07 | 0.00 | 0.09 | **0.57** |
| item23 | -0.01 | **0.34** | -0.09 | 0.03 | 0.12 | 0.09 |
| *item24* | 0.13 | 0.25 | -0.09 | -0.03 | -0.05 | 0.07 |

**Note:** items shown in italic were deleted in further analysis due to weak loading or cross loading.

**Supplementary results of Study 2**

*Independent behavioral study*

We conducted an independent behavioral study in which participants (N = 44, 32 males, M_age_ = 22.2 yrs) were asked to make punishment decisions on both same-race and other-race targets in a conflict context similar to that in the behavioral test reported in the main text of our paper. The sample size was similar to previous research investigating the behavioral and neural mechanisms of outgroup punishment under intergroup conflict situations (Han et al., 2020). In this independent study, either a White (Experimental condition) or an Asian (Control condition) played the role of an aggressor to harm an Asian target in the high and low conflict conditions. The participants were asked to choose the level of electrical shock to punish the Asian and White aggressors. The measure of punishment decisions in high vs. low conflict conditions was designed to control individual differences in context-independent tendencies to give strong (or weak) punishment to others. Comparing this measure related to White and Asian targets allowed us to examine the difference of punishment decisions towards same-race and other-race targets. A repeated-measures ANOVA with Race (White vs. Asian target) and Condition (high vs. low conflict) as within-subjects variables revealed a significant interaction (*F*(1,43) = 7.83, *p* = 0.008, Figure S2), suggesting that that the participants punished more harshly towards racial outgroup compared to racial ingroup targets in a similar conflict context.


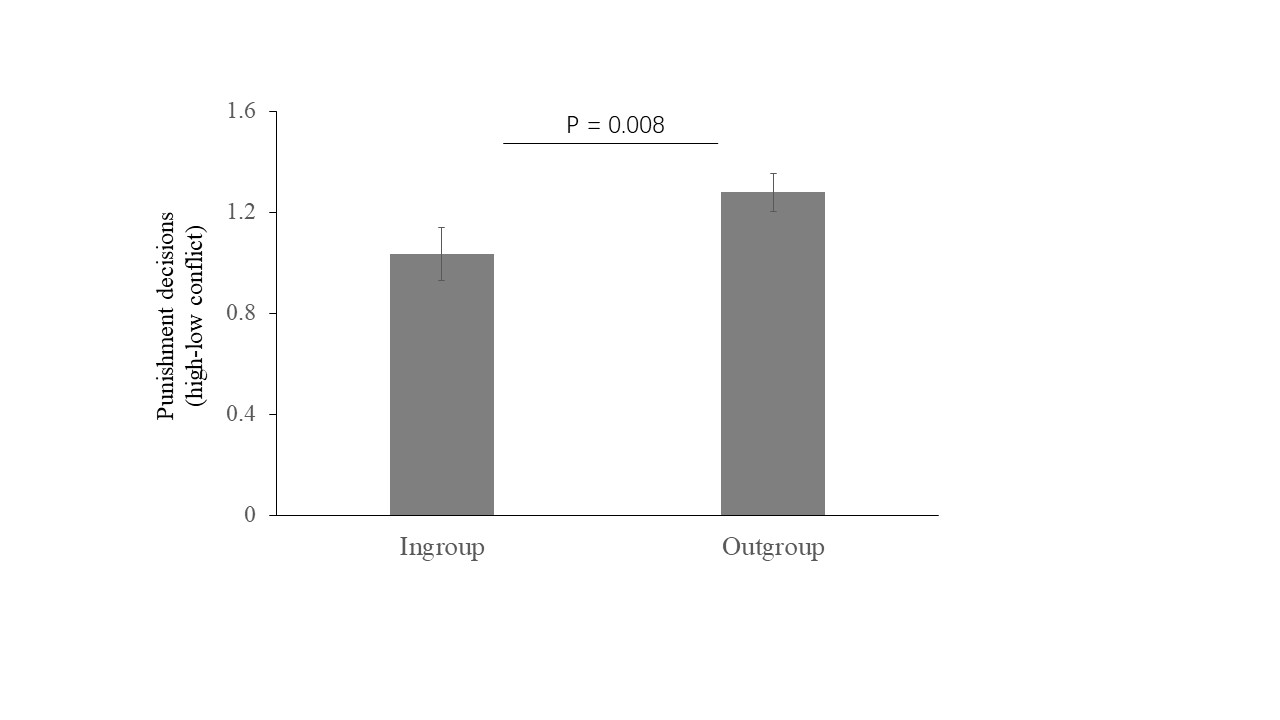


**Figure S2.** The punishment decisions to ingroup and outgroup in the independent study.


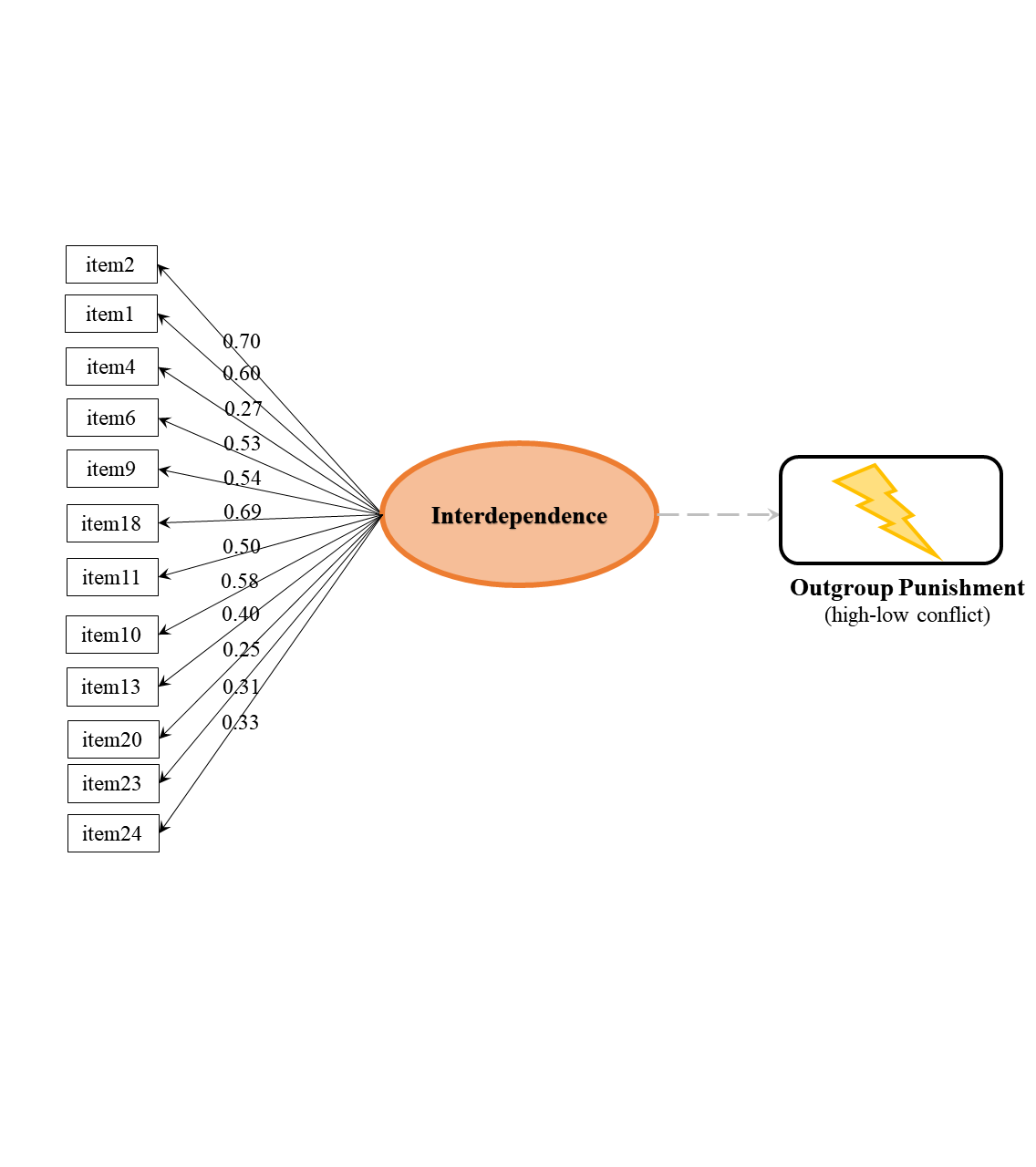


**Figure S3. Alternative SEM models relating interdependence to outgroup punishment decisions.** The gray dashed line shows the insignificant parameter. All parameter estimates shown are fully standardized. This model does not fit the data well (χ2 = 499.59, df = 65, p < 0.001, RMSEA = 0.100 (0.091–0.108), CFI = 0.839, SRMR = 0.076).


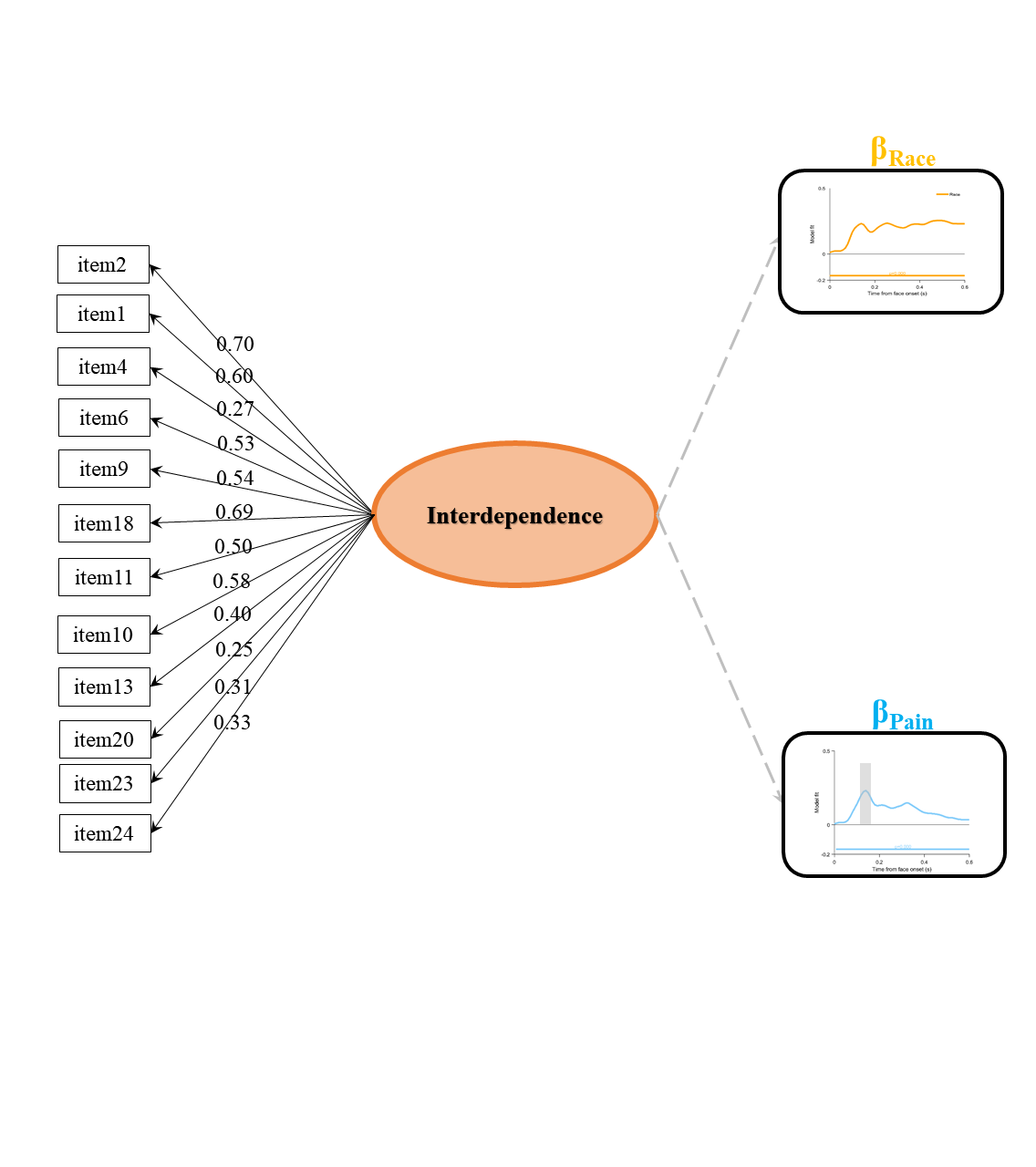


**Figure S4. Alternative SEM models relating interdependence to early neural representation to race and pain information.** The gray dashed lines show the insignificant parameters. All parameter estimates shown are fully standardized. This model does not fit the data well (χ2 = 495.220, df = 76, p < 0.001, RMSEA = 0.090 (0.083–0.098), CFI = 0.846, SRMR = 0.070).


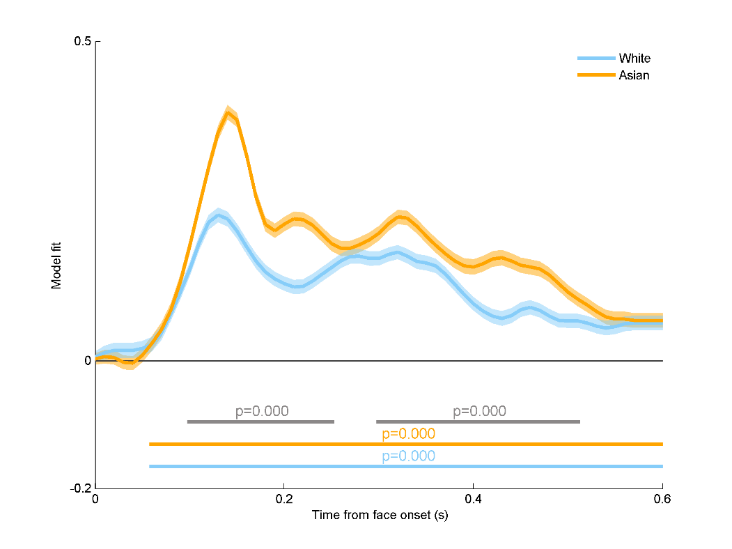


**Figure S5.** The time courses of the neural representation of pain, separately for Asian (orange line) and White (blue line) faces. The orange and blue lines below the plot indicate significant times, and the grey lines indicate significant differences between conditions both using cluster-based sign permutation test (cluster-defining threshold p < 0.05, and corrected significance level p < 0.05).

**Table S2.** Correlations between the neural representation of pain (separate the Asian and White condition) and punishment towards the outgroup.

|  | 120-160 ms | | 100–250 ms | | 300-510 ms | |
| --- | --- | --- | --- | --- | --- | --- |
|  | Asian | White | Asian | White | Asian | White |
| Outgroup punishment  (high – low conflict) | r = 0.052 | r = 0.020 | r = 0.008 | r = 0.053 | r = 0.013 | r = 0.065 |
|  | p = 0.180 | p = 0.601 | p = 0.883 | p = 0.166 | p = 0.740 | p = 0.090 |

Note: 120-160 ms corresponds to the time window showed the peak of neural representation of pain. 100-250 ms and 300-510 ms correspond to the time window showed greater neural representation of pain for Asian compared to White faces.

**Table S3.** Sampling variability of punishment-related correlation coefficents in each sampling bin

| **EG & Punishment** | | | | | |
| --- | --- | --- | --- | --- | --- |
| **Sample**  **size** | **Mean**  **coefficient** | **95% CI**  **(lower)** | **95% CI**  **(upper)** | **99% CI**  **(lower)** | **99% CI (upper)** |
| 25 | 0.10 | -0.32 | 0.52 | -0.43 | 0.58 |
| 29 | 0.10 | -0.28 | 0.47 | -0.33 | 0.50 |
| 35 | 0.11 | -0.27 | 0.43 | -0.34 | 0.48 |
| 42 | 0.11 | -0.21 | 0.41 | -0.27 | 0.45 |
| 50 | 0.11 | -0.19 | 0.38 | -0.25 | 0.43 |
| 59 | 0.10 | -0.16 | 0.36 | -0.25 | 0.40 |
| 71 | 0.10 | -0.14 | 0.34 | -0.20 | 0.37 |
| 84 | 0.11 | -0.11 | 0.31 | -0.15 | 0.36 |
| 100 | 0.11 | -0.12 | 0.32 | -0.16 | 0.36 |
| 119 | 0.10 | -0.08 | 0.29 | -0.13 | 0.33 |
| 142 | 0.10 | -0.09 | 0.27 | -0.13 | 0.31 |
| 169 | 0.11 | -0.05 | 0.26 | -0.09 | 0.29 |
| 200 | 0.10 | -0.05 | 0.24 | -0.08 | 0.26 |
| 239 | 0.10 | -0.04 | 0.24 | -0.07 | 0.27 |
| 284 | 0.10 | -0.02 | 0.23 | -0.04 | 0.25 |
| 338 | 0.10 | -0.01 | 0.22 | -0.03 | 0.24 |
| 401 | 0.10 | 0.00 | 0.20 | -0.02 | 0.22 |
| 478 | 0.10 | 0.00 | 0.19 | -0.01 | 0.20 |
| 568 | 0.10 | 0.02 | 0.19 | 0.00 | 0.20 |
| 676 | 0.10 | 0.03 | 0.18 | 0.01 | 0.19 |
| **RI & Punishment** | | | | | |
| **Sample**  **size** | **Mean**  **coefficient** | **95% CI**  **(lower)** | **95% CI**  **(upper)** | **99% CI**  **(lower)** | **99% CI (upper)** |
| 25 | -0.12 | -0.53 | 0.30 | -0.60 | 0.39 |
| 29 | -0.12 | -0.49 | 0.26 | -0.54 | 0.33 |
| 35 | -0.12 | -0.45 | 0.22 | -0.51 | 0.26 |
| 42 | -0.12 | -0.42 | 0.20 | -0.48 | 0.27 |
| 50 | -0.11 | -0.42 | 0.17 | -0.47 | 0.21 |
| 59 | -0.12 | -0.37 | 0.15 | -0.43 | 0.21 |
| 71 | -0.11 | -0.36 | 0.14 | -0.39 | 0.17 |
| 84 | -0.12 | -0.34 | 0.13 | -0.38 | 0.16 |
| 100 | -0.12 | -0.33 | 0.09 | -0.35 | 0.14 |
| 119 | -0.11 | -0.30 | 0.08 | -0.34 | 0.11 |
| 142 | -0.11 | -0.28 | 0.06 | -0.31 | 0.08 |
| 169 | -0.12 | -0.28 | 0.05 | -0.31 | 0.08 |
| 200 | -0.12 | -0.25 | 0.03 | -0.29 | 0.05 |
| 239 | -0.11 | -0.25 | 0.03 | -0.28 | 0.05 |
| 284 | -0.12 | -0.23 | 0.00 | -0.26 | 0.02 |
| 338 | -0.12 | -0.23 | 0.00 | -0.26 | 0.01 |
| 401 | -0.12 | -0.22 | -0.01 | -0.23 | 0.01 |
| 478 | -0.11 | -0.21 | -0.02 | -0.22 | 0.00 |
| 568 | -0.11 | -0.20 | -0.03 | -0.21 | -0.01 |
| 676 | -0.11 | -0.19 | -0.04 | -0.21 | -0.02 |

EG: Esteem for group; RI: Relational interdependence

**Table S4.** Sampling variability of race-related correlation coefficents in each sampling bin

| **EG & Race** | | | | | |
| --- | --- | --- | --- | --- | --- |
| **Sample**  **size** | **Mean**  **coefficient** | **95% CI**  **(lower)** | **95% CI**  **(upper)** | **99% CI**  **(lower)** | **99% CI (upper)** |
| 25 | 0.10 | -0.31 | 0.49 | -0.40 | 0.53 |
| 29 | 0.11 | -0.26 | 0.46 | -0.35 | 0.49 |
| 35 | 0.10 | -0.25 | 0.43 | -0.35 | 0.48 |
| 42 | 0.10 | -0.23 | 0.39 | -0.28 | 0.44 |
| 50 | 0.10 | -0.18 | 0.37 | -0.23 | 0.41 |
| 59 | 0.09 | -0.19 | 0.34 | -0.24 | 0.37 |
| 71 | 0.10 | -0.14 | 0.33 | -0.20 | 0.36 |
| 84 | 0.10 | -0.12 | 0.32 | -0.18 | 0.37 |
| 100 | 0.10 | -0.11 | 0.30 | -0.14 | 0.34 |
| 119 | 0.10 | -0.10 | 0.27 | -0.13 | 0.29 |
| 142 | 0.11 | -0.07 | 0.27 | -0.09 | 0.29 |
| 169 | 0.10 | -0.06 | 0.26 | -0.09 | 0.29 |
| 200 | 0.10 | -0.04 | 0.24 | -0.08 | 0.28 |
| 239 | 0.10 | -0.03 | 0.24 | -0.06 | 0.26 |
| 284 | 0.10 | -0.02 | 0.23 | -0.03 | 0.25 |
| 338 | 0.11 | -0.01 | 0.21 | -0.03 | 0.22 |
| 401 | 0.10 | 0.00 | 0.20 | -0.02 | 0.22 |
| 478 | 0.10 | 0.01 | 0.19 | -0.01 | 0.21 |
| 568 | 0.10 | 0.03 | 0.19 | 0.01 | 0.21 |
| 676 | 0.10 | 0.03 | 0.18 | 0.01 | 0.20 |
| **RI & Race** | | | | | |
| **Sample**  **size** | **Mean**  **coefficient** | **95% CI**  **(lower)** | **95% CI**  **(upper)** | **99% CI**  **(lower)** | **99% CI (upper)** |
| 25 | -0.09 | -0.47 | 0.30 | -0.53 | 0.38 |
| 29 | -0.08 | -0.46 | 0.29 | -0.52 | 0.35 |
| 35 | -0.09 | -0.41 | 0.25 | -0.45 | 0.28 |
| 42 | -0.08 | -0.39 | 0.22 | -0.44 | 0.29 |
| 50 | -0.09 | -0.36 | 0.20 | -0.41 | 0.27 |
| 59 | -0.08 | -0.33 | 0.16 | -0.39 | 0.22 |
| 71 | -0.08 | -0.32 | 0.16 | -0.37 | 0.20 |
| 84 | -0.09 | -0.30 | 0.12 | -0.37 | 0.17 |
| 100 | -0.09 | -0.27 | 0.10 | -0.31 | 0.13 |
| 119 | -0.08 | -0.25 | 0.09 | -0.29 | 0.12 |
| 142 | -0.09 | -0.25 | 0.07 | -0.28 | 0.11 |
| 169 | -0.09 | -0.24 | 0.07 | -0.25 | 0.10 |
| 200 | -0.09 | -0.23 | 0.05 | -0.26 | 0.08 |
| 239 | -0.09 | -0.21 | 0.04 | -0.25 | 0.08 |
| 284 | -0.08 | -0.21 | 0.03 | -0.23 | 0.06 |
| 338 | -0.09 | -0.19 | 0.02 | -0.22 | 0.05 |
| 401 | -0.09 | -0.18 | 0.01 | -0.20 | 0.03 |
| 478 | -0.08 | -0.17 | 0.00 | -0.20 | 0.02 |
| 568 | -0.09 | -0.17 | -0.01 | -0.19 | 0.00 |
| 676 | -0.09 | -0.16 | -0.01 | -0.18 | 0.01 |

EG: Esteem for group; RI: Relational interdependence

**References:**

Bentler PM, & Bonett DG. 1980. Significance tests and goodness of fit in the analysis of covariance structures. *Psychol Bull.* 88: 588.

Hardin EE, Leong FT, & Bhagwat AA. 2004. Factor structure of the self-construal scale revisited: Implications for the multidimensionality of self-construal. *J Cross Cult Psychol,* 35: 327-345.

Hu Lt., & Bentler PM. 1999. Cutoff criteria for fit indexes in covariance structure analysis: Conventional criteria versus new alternatives. *Struct Equ Modeling: a Multidisciplinary Journal.* 6: 1-55.

Pagliaccio D, Luking KR, Anokhin AP, Gotlib IH, Hayden EP, Olino TM, Peng CZ, Hajcak G, & Barch DM. 2016. Revising the BIS/BAS Scale to study development: Measurement invariance and normative effects of age and sex from childhood through adulthood. *Psychol Assess.* 28: 429.
